# On‐Chip Chemiresistive Sensor Array for On‐Road NO*_x_* Monitoring with Quantification

**DOI:** 10.1002/advs.202002014

**Published:** 2020-09-30

**Authors:** Hi Gyu Moon, Youngmo Jung, Beomju Shin, Young Geun Song, Jae Hun Kim, Taikjin Lee, Seok Lee, Seong Chan Jun, Richard B. Kaner, Chong‐Yun Kang, Chulki Kim

**Affiliations:** ^1^ National Center for Efficacy Evaluation of Respiratory Disease Product Korea Institute of Toxicology Jeongeup Jeollabuk‐do 56212 Republic of Korea; ^2^ Center for Electronic Materials Korea Institute of Science and Technology (KIST) Seoul 02792 Republic of Korea; ^3^ Department of Chemistry and Biochemistry University of California Los Angeles CA 90095 USA; ^4^ Sensor System Research Center Korea Institute of Science and Technology (KIST) Seoul 02792 Republic of Korea; ^5^ Department of Material Science and Engineering Yonsei University Seoul 03722 Republic of Korea; ^6^ Department of Materials Science and Engineering University of California Los Angeles CA 90095 USA; ^7^ KU‐KIST Graduate School of Converging Science and Technology Korea University Seoul 02841 Republic of Korea

**Keywords:** chemiresistive sensor arrays, Langmuir isotherms, metal oxide semiconductors, NO*_x_* sensors

## Abstract

The adverse effects of air pollution on respiratory health make air quality monitoring with high spatial and temporal resolutions essential especially in cities. Despite considerable interest and efforts, the application of various types of sensors is considered immature owing to insufficient sensitivity and cross‐interference under ambient conditions. Here, a fully integrated chemiresistive sensor array (CSA) with parts‐per‐trillion sensitivity is demonstrated with its application for on‐road NO*_x_* monitoring. An analytical model is suggested to describe the kinetics of the sensor responses and quantify molecular binding affinities. Finally, the full characterization of the system is connected to implement on‐road measurements on NO*_x_* vapor with quantification as its ultimate field application. The obtained results suggest that the CSA shows potential as an essential unit to realize an air‐quality monitoring network with high spatial and temporal resolutions.

## Graphical abstract

A chemiresistive sensor array (CSA) is realized by conventional semiconductor fabrication processes in the wafer‐scale. The CSA demonstrates outstanding performance in terms of sensitivity, selectivity, and stability. The CSA with plug and play capability is integrated with signal‐processing and wireless‐communication modules. To accurately quantify the NO*_x_* concentration, the Langmuir isotherm model is applied. These advancements enable on‐road NO*_x_* monitoring with the CSA.

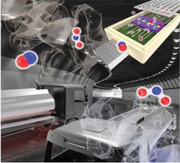

## 1 Introduction

When N_2_ is released during a combustion process, it combines with oxygen atoms to create nitric oxide (NO). This further combines with O_2_ to create nitrogen dioxide (NO_2_), and collectively, NO and NO_2_ are referred to as nitrogen oxides (NO*_x_*).^[^ ^1^, ^2^, ^3^, ^4^ ^]^ Various human activities with motor vehicles, electric utilities, and industrial boiling machines increase the levels of NO*_x_* in the atmosphere.^[^ ^5^ ^]^ NO*_x_* gases react, forming smog and acid rain, and are central to the formation of fine particles and ground‐level ozone.^[^ ^6^, ^7^ ^]^ This significantly impacts respiratory conditions, causing inflammation of airways at a high level.^[^ ^8^, ^9^, ^10^, ^11^, ^12^ ^]^ Although a variety of measures have been proposed to monitor the sources of air pollution, monitoring with high spatial resolution and quantification capability still remains as a chronic challenge.^[^ ^13^, ^14^, ^15^, ^16^ ^]^

In the sense that semiconductor gas sensors can be manufactured on a microchip scale and deployed with high density, a network of microair quality sensors is expected to provide a spatially resolved map of air pollution.^[^ ^17^, ^18^, ^19^ ^]^ One of highly promising candidates for this application is metal‐oxide semiconductor (MOS) sensor arrays. MOS sensor arrays have been demonstrated to detect a variety of gas analytes with sensitivities of parts per billions (ppb) and developed into integrated systems for various purposes.^[^ ^20^, ^21^, ^22^, ^23^, ^24^, ^25^ ^]^ They have also demonstrated the capability of recognizing response patterns of analytes leading to effective improvement in selectivity.^[^ ^26^, ^27^ ^]^ However, most of them have been focused on reducing the detection limit, with little effort expended on quantifying their kinetic responses based on the binding affinities and miniaturizing the entire system in which the active sensors are typically required to operate at high temperature. Additionally, the inherent variability between sensors and baseline drift often lead to difficulty in statistical analysis of the sensor responses. Although the integration of multiple sensors array in wafer‐scale has been endeavored, difficulties in large‐scale fabrication for commercialization still remain.^[^ ^28^, ^29^, ^30^, ^31^, ^32^, ^33^ ^]^ Here, we fabricated a fully integrated chemiresistive sensor array (CSA) consisting of 16 sensor elements by facilitating conventional semiconductor manufacturing processes for micro‐ and nanoscale patterning. This approach provides a sensor platform desirable for mass‐production, reduces inherent variability between devices and allows for statistical analysis for obtained signals. Collection of sensor responses in the array configuration improves selectivity via the application of pattern recognition algorithm. The binding affinity of NO*_x_* vapor molecules is determined by adopting the Langmuir isotherm model. This enables accurate signal to analyte concentration conversion. To miniaturize the entire system, we carefully designed the circuit board to thermally isolate electrical circuit elements from the heated sensor array. Finally, we combined all these ingredients to demonstrate on‐road NO*_x_* monitoring in urban environment as an ultimate field application.

## 2 Results and Discussion

**Figure 1:**
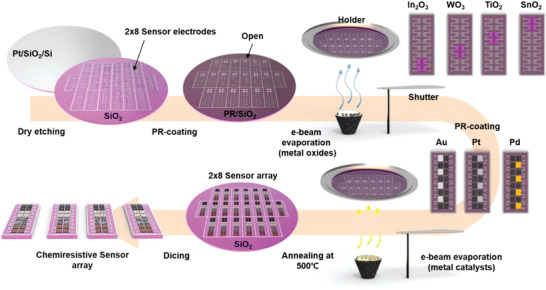
Schematic diagram of wafer‐scale fabrication processes of chemiresistive sensor array (CSA) with 16 sensor elements.

To minimize the variability in device performance, sequential semiconductor fabrication processes were applied to realize 16 sensor elements in our CSA as illustrated in **Figure** **1** (Figure S1AB, Supporting Information).^[^ ^34^, ^35^ ^]^ Pt interdigitated electrodes are fabricated on the SiO_2_/Si substrates with microelectro mechanical system (MEMS) technologies such as photolithography, e‐beam evaporation, and lift‐off. Deposition of four different metal oxides (SnO_2_, TiO_2_, WO_3_, and In_2_O_3_) onto predefined patterns is followed facilitating sequential photolithography processes and electron beam evaporation. The CSA with 16 sensor elements was functionalized by depositing 3 nm thick novel metals (Au, Pt, and Pd). The thin films in 16 sensor elements are obtained to be 100 nm thick with the roughness of 3 to 20 nm, as described in Figures S2 and S3 (Supporting Information). The fabricated CSA was finally annealed at 500 °C in air for 2 h to crystallize the metal oxide thin films, simultaneously forming Au islands in nanoscale via the agglomeration of the Au films. The WO_3_, SnO_2_, TiO_2_, and In_2_O_3_ thin films were characterized by x‐ray diffraction (XRD), revealing that the as‐deposited amorphous films were crystallized to cassiterite crystal phase with the tetragonal rutile structure of SnO_2_, the polycrystalline anatase phase of TiO_2_, the monoclinic phase of WO_3_, the cubic bixbyite structure of In_2_O_3_ (Figure S4, Supporting Information). No characteristic feature due to the existence of the Au catalyst was observed for their random distribution. The detailed fabrication processes are described in Experimental Section.

The key achievement from the design of the overall system was to optimize the capability of the CSA in terms of sensitivity, selectivity and reproducibility. In previous works, we observed that the sensor responses were increased with the deposition of an Au layer.^[^ ^36^, ^37^, ^38^ ^]^ It was found that the enhancement was maximized at the Au layer thickness of 3 nm. With other thicknesses of the Au layer of 1, 5, and 10 nm, those formed Au nanoparticles (NPs) provided weaker catalytic effect and lower sensor responses by the interference of adsorbed gas molecules. On the other hand, in signal processing, different ranges of the resistance variation of the applied sensor elements degrade its ability to distinguish target analytes. It is because all the sensor elements share the same 16‐bit ADC where the digitalized signal resolution depends linearly on the total range of the resistance values of all the sensors. And this severely harms the selectivity of the CSA. Hence, we designed the configuration of the sensor array in the manner that they have the resistance variation for targeted reactions in the desired range and the obtained responses are located in the PCA with maximum separation. Finally, our approach with the array configuration is based on the principle that the collection of responses from different sensor elements improves the selectivity for target analytes. To evaluate this, we investigated the dependence of the classification accuracy on the array configuration for individual vapors and vapor mixtures. It is seen that our CSA comprising of 16 sensor elements has higher classification accuracy (with the application of the SVM classifier) with the increased number of target analytes and demonstrates better reproducibility (or less standard deviation in the obtained responses) than 2 × 2 and 3 × 3 sensor arrays which consist of metal oxide films, the same films with catalysts and nanostructured metal oxides (Figure S5A,B, Supporting Information). The relatively large standard deviation in the response amplitudes of the nanostructured MOS sensors is attributed to its strong dependence on the environment such as temperature and humidity.

**Figure 2:**
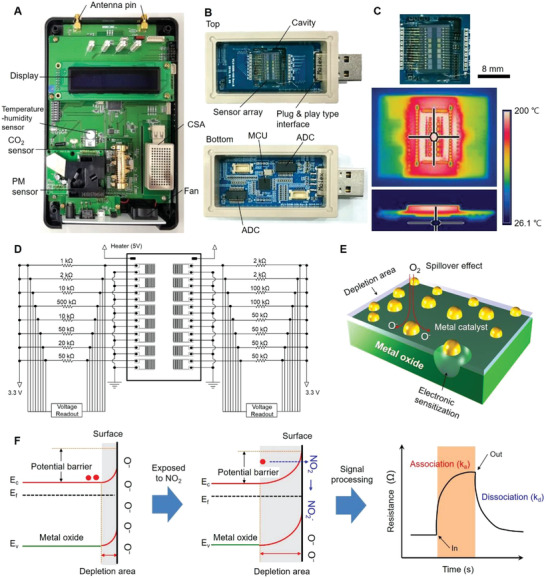
Fully integrated measurement system and NO_2_ sensing mechanism. A) A photographic image of the fully integrated system. B) Photographs of the upper and lower circuit boards in the CSA module with plug and play capability. C) Thermographic images (top and side views) of the Au‐wire bonded CSA. D) Schematic of electrical connections for multiplexed signal acquisition. E) Schematic of the enhanced sensing mechanism by metallic catalysts (10–20 nm). F) Band structure diagrams of the sensing principle based on the change in the electrical conductivity of the MOS thin film upon exposure to NO_2_. For n‐type metal oxides, oxidizing gas (acceptor) increases the resistance of the thin film.

A fully integrated system is primarily composed of a CSA module, commercialized sensors (temperature‐humidity, CO_2_, and particulate matters (PMs)), signal‐processing circuitry, and a wireless‐communication module (**Figure** **2**A). The system level overview of the signal transduction, conditioning, processing, and wireless transmission is described in Figure S6 (Supporting Information). The CSA module has plug and play capability (Figure 2B) in which all the sensor elements in the CSA module underwent an aging process for 72 h to make the sensing materials thermally stable. Since metal‐oxide semiconductor sensors operate at a typical high temperature of >150 °C, isolation of the active sensing area from the rest of the circuitry is required for reliable sensing and signal processing. For this, a sensor array and a heater unit are placed in a separate board in which the periphery of the active sensing area is cut out to minimize heat transfer. The realized local heating is manifested in the false‐colored thermal image of the integrated board (Figure 2C). Figure 2D schematically shows the connections for multiplexed signal acquisition on the upper board of the CSA module (Figure S7, Supporting Information). A 16‐bit analog‐to‐digital converter (ADC) and miscellaneous circuit components are configured such that the final analogue output of each sensor is finely resolved. The microcontroller's computational capability is used to compensate and relay the sensor response signals to a gateway. The gateway facilitates wireless data transmission to upload the data to a server for post‐processing and sharing. Figure 2E illustrates the sensing layer and deposited functional catalysts (Au, Pd, and Pt) with diameters of ≈10–20 nm. The crystallinity of the thermally deposited thin films and the functionality of the metallic catalysts play major roles in sensing capability. Nanograin boundaries formed after calcination lead to the formation of double Schottky barriers which enhance the sensor responses by expanding the depletion region.^[^ ^24^, ^39^ ^]^ The response to NO_2_ vapor at relatively low temperature (about 180 °C) is observed due to the stronger oxidizing agent (NO_2_^−^) than ionized oxygen species. The resistance of sensor increases due to the expansion of depletion area by chemicaptured electrons of NO_2_, Therefore, trapped electrons from NO_2_ molecules and induced band bending are mainly responsible for the conduction change (Figure 2F).^[^ ^40^ ^]^

**Figure 3:**
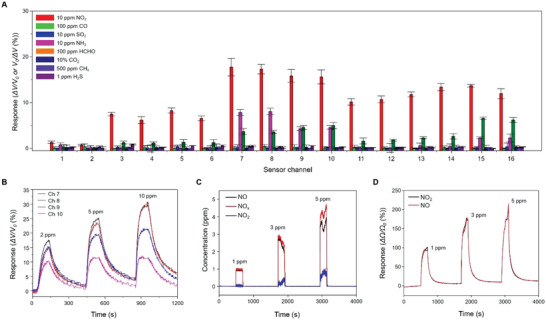
Highly selective sensing of NO*_x_*. A) Sensing capability of the CSA for different chemical vapors including 10% CO_2_; 100 parts per million (ppm) HCHO and CO; and10 ppm NH_3_ and SO_2_; 500 ppm CH_4_; 1 ppm H_2_S and 10 ppm NO_2_. B) Representative responses of Ch 7–10 to NO_2_ vapors at concentrations of 2–10 ppm. C) Responses of a high‐precision NO*_x_* analyzer to NO vapor. It is noted that a fraction of the NO vapor was converted into NO_2_ vapor. D) Responses of the CSA to NO and NO_2_ vapors at concentrations of 1, 3, and 5 ppm.

To evaluate the sensing capability of the CSA, it was placed in a carefully designed chamber. Since the relative humidity and temperature conditions are critical for reliable data acquisition, they were consistently monitored through the entire measurements using a thermohydrometer (Dwyer, Model RP2 Thermo‐Hygrometer Probe). The total vapor flow was kept at 2000 sccm cm^3^ min^−1^ by using a programmable mass flow controller (MFC). Details of the gas control system and the characterization chamber are described in Figures S8 and S9 (Supporting Information). The sensing capability of the CSA was evaluated under exposure to different chemical vapors, including 10% CO_2_; 100 parts per million (ppm) HCHO and CO; and10 ppm NH_3_ and SO_2_; 500 ppm CH_4_; 1 ppm H_2_S and 10 ppm NO_2_ (**Figure** **3**A and Figure S10, Supporting Information). The obtained responses were normalized as *R* = (*V*_gas_ ‐ *V*_0_)/*V*_0_ × 100 (%) for oxidizing gases and *R* = (*V*_0_ ‐ *V*_gas_)/*V*_0_ × 100 (%) for reducing gases, where *V*_0_ and *V*_gas_ denote the initial voltage of the sensor in air and the obtained voltage in response to the analyte. The CSA exhibited outstanding sensitivity and selectivity to NO_2_ vapor against other chemical vapors. The enhanced response to NO_2_ vapor is attributed to the dominant presence of an oxidizing agent (NO_2_^−^) at a relatively low temperature. Figure 3B shows the representative responses of Ch 7–10 to NO_2_ vapor at concentrations of 2–10 ppm (Figure S11, Supporting Information). The theoretical detection limit for NO_2_ (except for Ch 1 and 2) was evaluated to be in the range of 609–896 ppt via linear extrapolation (Figure S12 and Table S1, Supporting Information).^[^ ^34^ ^]^ We experimentally demonstrated the detection of NO_2_ vapors at the ppb‐level concentrations using our CSA (Figure S13, Supporting Information). These sensing capabilities were maintained in a high humidity (relative humidity 80%) condition (Figure S14, Supporting Information). A long‐term stability test shows that the sensitivity of these gas sensors remains at the same level for 317 d (Figure S15, Supporting Information). Principal component analysis (PCA) results based on the normalized responses of 16 sensor elements are presented in Figure S16 (Supporting Information). NO_2_, NH_3_, and HCHO vapors are well grouped in the PCA plot in contrast to other vapors, which are overlapped with air. This indicates that deposited metallic nanoparticles (NPs) allowed for the enhancement of the selective gas detection via the catalytic sensitization and that the degree of selectivity depended on the configuration of the active sensing elements.

As a NO*_x_* analyzer, the response of the CSA to NO vapor should be characterized. However, as seen in Figure 3C, after NO vapor at different concentrations of 1, 3, and 5 ppm was introduced into a chamber, it was observed by a high‐precision apparatus (Thermo Fisher Scientific, Model 42) that a fraction of NO vapor was rapidly converted into NO_2_ vapor. This makes its precise quantification of the NO vapor concentration difficult under ambient conditions. For our CSA, different species of oxygen ions (including O_2_^−^, O^−^, and O_2_^−^) are formed on the surface of metal‐oxide sensors depending on the thermal energy conditions.^[^ ^37^ ^]^ Considering that the heater unit maintains the surface temperature of sensor elements at 180 °C, O^−^ and O_2_^−^ ions mainly exist on the surface. This allows for the following chemical reactions.^[^ ^41^ ^]^

(1) $$\begin{array}{c} NO_{2}\left(\mathrm{gas}\right)+e^{-}\to NO^{2-}\left(\mathrm{ads}\right) \end{array}$$

(2) $$\begin{array}{c} \mathrm{NO}\left(\mathrm{gas}\right)+e^{-}\to \;NO^{-}\left(\mathrm{ads}\right) \end{array}$$

From these, it is expected that both reactions of NO and NO_2_ vapors at a certain concentration remove the same number of electrons from the active sensing layer. And it was confirmed that they cause almost the same resistance changes in the response curves (Figure 3D and Figure S17, Supporting Information). Therefore, it is reasonable to apply the obtained NO_2_ conversion relationship for NO*_x_* analysis in the following measurements. These characteristics endow the CSA with a major advantage for NO*_x_* sensor applications.

**Figure 4:**
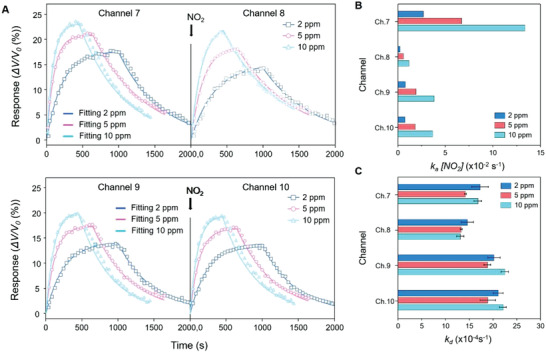
Quantification of the affinities of NO_2_ interactions using the CSA. A) Real‐time sensor responses and their fittings to the Langmuir isotherm model at the four channels (Ch. 7, 8, 9, and 10), where the timespan for each step was adjusted in such a manner that a sensor reached its own equilibrium state at different concentrations of NO_2_ vapors. B,C) Obtained association rate constant (*k*_a_) and dissociation rate constant (*k*_d_) for Ch. 7, 8, 9, and 10 (Table S2, Supporting Information).

**Figure** **4**A presents real‐time sensor responses with its fitting according to the Langmuir equation for Channel 7, 8, 9, and 10 where the time span for each step was adjusted in such a manner that a sensor reached its own equilibrium state at different concentrations of NO_2_ vapors.^[^ ^42^ ^]^ These channels were made of In_2_O_3_, which is known to have high selectivity to NO*_x_*. The CSA monitors voltage differences across external resistances connected to each element of the sensor array in series (Figure 2D). The voltage values directly reflect the resistance change of the corresponding sensors. The voltage change across the external resistance is described as (Experimental Section)

(3) $$\begin{array}{c} \Delta V\;=\frac{1.65}{\frac{2R_{F}}{C\left[AB\right]}-1} \end{array}$$

where $C\;=\;\frac{n_{a}L}{n_{S1}n_{S2}q\mu }$, with *n*_a_ being the electric surface charge density; *L* being the length of the sensing channel; *n*_S1_ and *n*_S2_ being the charge densities before and after the chemical vapor reaction, respectively; *q* being the carrier charge, and *μ*being the carrier mobility. [AB] is the number of chemical vapor molecules involved in the reactions. Assuming that the surface is homogeneous, [AB] is expressed for absorption and desorption processes as (Experimental Section):^[^ ^34^ ^]^

(4) $$\begin{array}{c} {\left[AB\right]}_{\mathrm{absorption}}=\frac{k_{a}{\left[B\right]}_{\max}\left[A\right]}{k_{a}\left[A\right]+k_{d}}\;\left(1-e^{-\left(k_{a}\left[A\right]+k_{d}\right)t}\right) \end{array}$$

(5) $$\begin{array}{c} {\left[AB\right]}_{desorption}=\frac{k_{a}{\left[B\right]}_{max}\left[A\right]}{k_{a}\left[A\right]+k_{d}}\;e^{-k_{d}t} \end{array}$$

Since the sensor responses of the CSA are traced in real time, both the binding constants and the rate constants can be determined by fitting the temporal response curve with the kinetic model described above. For instance, the obtained values of the association rate constant (*k*_a_) and dissociation rate constant (*k*_d_) for Channel 7 were *k*_a_ = 2.986 ± 0.6872 × 10^5^ M^−1^ s^−1^ and *k*_d_ = 16.1 ± 1.6643 × 10^−4^ s^−1^, respectively. The results for those four channels are summarized in Figure 4B,C and Table S2 (Supporting Information). The association constant (*K*_A_ = *k*_d_/*k*_a_) was calculated using these association and dissociation rate constants. These values agreed well with the literature.^[^ ^43^ ^]^ Their variation between sensors based on identical materials is attributed to the difference in the combined surface energies of the NPs (Au, Pt, and Pd) and the corresponding metal oxide layers.

**Figure 5:**
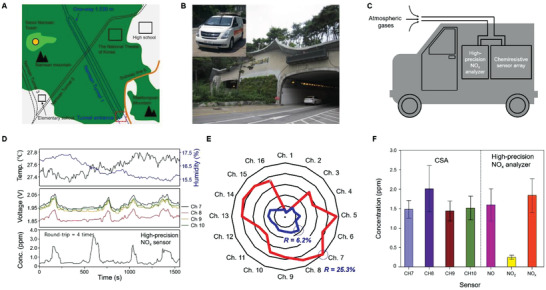
On‐road NO*_x_* monitoring with quantification. A) Schematic of on‐road measurement route (Namsan 1‐ho tunnel, 1.53 km, in Seoul). The red dashed box indicates one of the entrances. B) Photographs of the tunnel entrance and the vehicle designed for mobile air‐quality monitoring (inset). C) Schematic of air flow for measurement in the vehicle. D) Real‐time response curves of Ch. 7, 8, 9, and 10 for a high‐precision NO*_x_* analyzer and our CSA. E) Response patterns obtained in the tunnel measurement (blue) and 5 ppm NO*_x_* measurement in the laboratory chamber (red). F) Measured concentration chart for our CSA and the high‐precision NO*_x_* analyzer.

To demonstrate the ultimate applicability of our CSA, on‐road measurement on NO_x_ was performed on a route of 1.53 km (Namsan 1‐ho tunnel, Seoul) (**Figure** **5**A). Our CSA system was placed in a vehicle designed for mobile air‐quality monitoring (the inset of Figure 5B). This vehicle was equipped with different types of high‐precision gas and PM analyzers. The external air flow at the front of the vehicle was guided into a chamber connected to inlets of several air‐quality monitoring instruments (Figure 5C), minimizing the interference with the vehicle exhaust. Calibration was performed using high‐efficiency particulate air‐filtered air before all the measurements. The average temperature of tunnel during the measurements varied between 26 °C (entrance) and 30 °C (in the tunnel). The relative humidity was in the range of 15%–40% in the tunnel. Interference with vehicles traveling the opposite direction was minimized in the one‐way tunnel. Figure 5D shows the obtained response curve in the reaction and recovery phases for a commercial NO*_x_* analyzer (Thermo Fisher Scientific, Model 42) and our CSA. Rising peaks appeared corresponding to the responses at the moments at which the measurement system was collecting data in the tunnel. Time delay was observed between the measurements in the NO*_x_* analyzer and the CSA. This can be attributed to the different flow rates from a main chamber into the commercial NO*_x_* analyzer and the CSA.

The pattern of the responses of the fully integrated CSA (blue) clearly exhibited the same feature as the one of 5 ppm NO*_x_* responses in the laboratory chamber (red) (Figure 5E). This suggests possible library construction of target analyte fingerprints. By applying the reaction constants for concentration conversion, the responses of four channels (7–10) were calculated to be NO*_x_* concentrations of 1.5–2.3 ppm (Figure 5F). The average response of those four channels (7, 8, 9, and 10) in our CSA is 1.72 ppm. This agreed very well with the obtained average concentration of 1.78 ppm from the high‐precision instrument (Thermo Fisher Scientific, MA, USA; Model 42) within the range of one standard deviation.

## 3 Conclusion

We carefully designed a plug‐and‐play type sensor module comprising a fabricated CSA with operating circuitry and demonstrated its capability as low‐cost gas sensors for evaluating the spatial and temporal variation of NO*_x_*. We adopted a theoretical model to quantify the binding affinities in chemical vapor reactions and applied them for response to concentration conversion. Finally, we demonstrated the quantification of NO*_x_* vapor by performing on‐road measurement, and the comparison with the result obtained by a high‐precision instrument confirmed its validity. Application of conventional semiconductor manufacturing processes allows for its scalability and cost‐effective production. And this approach will minimize variability between devices and the scalable array configuration can maximize target‐specificity by the collection of different sensors’ responses and possible statistical analysis. With these advances, the CSA will bring us one step closer to the realization of the micro‐sensor network for air quality monitoring in high resolution.

## 4 Experimental Section

##### Fabrication and Integration

SiO_2_/Si substrates were patterned to define Pt interdigitated electrodes at a 4 in. wafer scale by using photolithography and dry etching (Oxford etcher) (Figure S1, Supporting Information). MOS thin films (100 nm thick) comprising WO_3_, SnO_2_, TiO_2_, and In_2_O_3_ were deposited onto predefined regions by using sequential processes of photolithography and in situ electron‐beam evaporation. After the deposition of 3 nm thick functionalizing layers with novel metals (Au, Pt, and Pd), the CSA with 16 sensor elements was realized. The base pressure and applied power for deposition were 2 × 10^−6^ mTorr and 50–70 kW, respectively. The deposition rate was 1.5–3.3 Å s^−1^. The fabricated CSA was annealed at 500 °C in air for 120 min to crystallize the amorphous metal‐oxide films, followed by an aging process for 72 h. After these thermal processes, the functionalized layer of the novel metals formed nanoscale metallic islands. Field‐emission scanning electron microscopy (FE‐SEM) images are shown in Figure S18 (Supporting Information). The fabricated CSA was wire‐bonded on a printed circuit board (PCB). Each sensor element was connected to an external resistor (*R*1 to *R*16) in series, and the voltages across the external resistors were recorded. The PCB was placed on a heater unit whose power consumption was 250 mW. This operation power per unit area of about 0.87 mW mm‐^2^ at 180 °C is comparably lower than other commercial gas sensors (Table S3, Supporting Information). The PCB was designed in the manner that the device was thermally isolated from other circuit components. The PCB with the CSA was plugged into a bottom board containing a microcontroller unit and a 16‐bit ADC for signal processing.

##### Morphology Imaging

The morphology of the deposited materials was investigated by using FE‐SEM (SU‐70, Hitachi) with 2‐nm Pt sputtering (except for Au, Pt, and Pd‐functionalized thin films). Thermographic and optical images of the CSA were obtained by using an infrared camera (SC660, FLIR) and an optical microscope (BX41M‐LED, Olympus Upright Microscope).

##### Real‐Time Sensor Response Fitting

As shown in Figure 2B, each sensor element was connected to an external resistor RF in series, and the voltage across it was measured. With the application of a total voltage of 3.3 V, the voltage difference across RF before and after chemical vapor reaction is described as:

(6) $$\begin{array}{c} \begin{array}{cc} \Delta V & =V_{2}-\;V_{1}=3.3\;R_{F}\;\left(\frac{1}{R_{F}+R_{S2}}-\;\frac{1}{R_{F}+R_{S1}}\right)\\ & =\;-1.65\frac{\Delta R}{2R_{F}+\Delta R}\;\mathrm{where}\;\Delta R=R_{S2}-R_{S1} \end{array} \end{array}$$

where Δ*R* = *R*_S2_ ‐ *R*_S1_, RF is the external resistance, *R*_S1_ and R_S2_ are the sensor resistance values before and after chemical vapor reaction. According to the Drude model, the resistance change before and after the chemical vapor sensing can be described as:

(7) $$\begin{array}{c} \Delta R\;=\frac{L}{\Delta nq\mu S}\;\;\mathrm{where}\;\Delta n\;=\;n_{a}S_{a}\left[AB\right] \end{array}$$

where *n_a_* is the electric charge transferred on a unit surface, and *S_a_* is the sensing area. With these, the voltage change across the sensor is described as:

(8) $$\begin{array}{c} \Delta V\;=\frac{1.65}{\frac{2R_{F}}{C\left[AB\right]}-1} \end{array}$$

Then, the Langmuir isotherm model was adopted for [*AB*] in adsorption and desorption processes as described in the following:

(9) $$\begin{array}{c} {\left[AB\right]}_{\mathrm{absorption}}=\frac{k_{a}{\left[B\right]}_{\max}\left[A\right]}{k_{a}\left[A\right]+k_{d}}\;\left(1-e^{-\left(k_{a}\left[A\right]+k_{d}\right)t}\right) \end{array}$$

(10) $$\begin{array}{c} {\left[AB\right]}_{\mathrm{desorption}}=\frac{k_{a}{\left[B\right]}_{\max}\left[A\right]}{k_{a}\left[A\right]+k_{d}}\;e^{-k_{d}t} \end{array}$$

where *k*_a_ and *k*_d_ are the association rate constant and dissociation rate constant, respectively; [*A*] is the concentration of chemical vapor; and [*B*]max is the maximum number of binding sites per unit area.

##### Measurement Setup

The USB‐type CSA module was placed in a chamber (16 × 16 × 50 cm^3^) volume for testing gas responses. The entire system, including pipelines, a mixing chamber, and the reaction chamber, was controlled under regulated conditions in terms of the temperature and relative humidity. The gas flow was controlled by an automated mass flow controller (MFC), where the total gas flow rate was kept at 2000 cm^3^ min^−1^. The sensor responses were measured to 500 ppm CO vapor according to various MFC flow rates ranging from 100 to 1000 cm^3^ min^−1^. It turned out that the flow rate did not have a critical effect on the sensitivity as shown in Figure S19 (Supporting Information). Considering the chamber volume for the measurement system with the fully integrated CSA, the flow rate of 2000 cm^3^ min^−1^ was used. The environmental conditions were monitored by a digital humidity and temperature meter (SMART SENSOR, AR837) during measurements.

## Conflict of Interest

The authors declare no conflict of interest.

## Supporting information

Supporting Information
