# Supplementary material for: On‐Chip Chemiresistive Sensor Array for On‐Road NOx Monitoring with Quantification
Source: Adv Sci (Weinh). 2020 Sep 30;7(22):2002014. doi: 10.1002/advs.202002014 (PMC7675194; doi:10.1002/advs.202002014)
Supplement: Supplementary file 1 — Supporting Information [file ADVS-7-2002014-s001.pdf]

© 2020 Wiley-VCH GmbH

ADVANCED SCIENCE
Open Access

Supporting Information

for *Adv. Sci.*, DOI: 10.1002/advs.202002014

On-Chip Chemiresistive Sensor Array for On-Road NOx Monitoring with Quantification

Hi Gyu Moon, Youngmo Jung, Beomju Shin, Jae Hun Kim, Taikjin Lee, Seok Lee, Seong Chan Jun, Richard B. Kaner, Chong-Yun Kang,* and Chulki Kim**

Supporting Information

On-chip chemiresistive sensor array for on-road NO_x monitoring with quantification

Hi Gyu Moon, Youngmo Jung, Beomju Shin, Jae Hun Kim, Taikjin Lee, Seok Lee, Seong Chan Jun, Richard B. Kaner, Chong-Yun Kang*, and Chulki Kim**

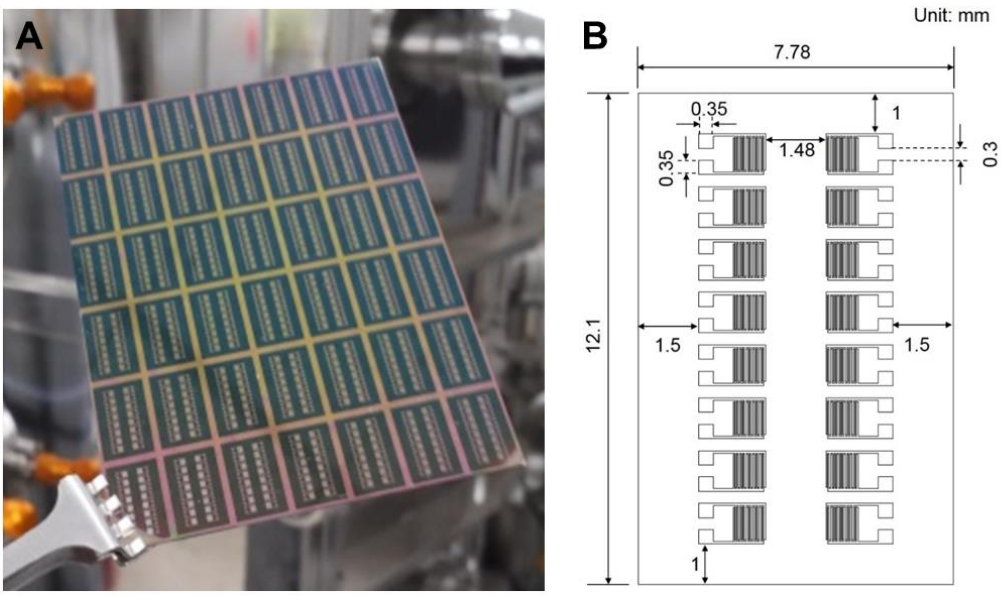

Figure S1. Wafer-scale fabrication of the chemiresistive sensor array (CSA) with 16 sensor elements. (A) Photograph image of fabricated CSAs in a wafer scale. (B) Dimension of the single CSA. 16 sensor elements with Pt interdigitated electrodes were patterned.

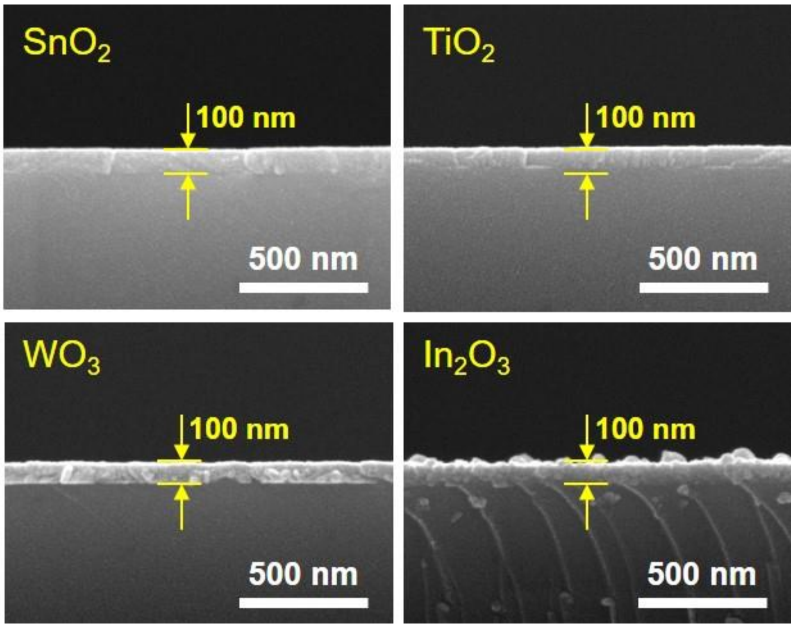

Figure S2. Cross-sectional FE-SEM images of four metal oxide (SnO_2, TiO_2, WO_3 and In_2O_3) thin films.

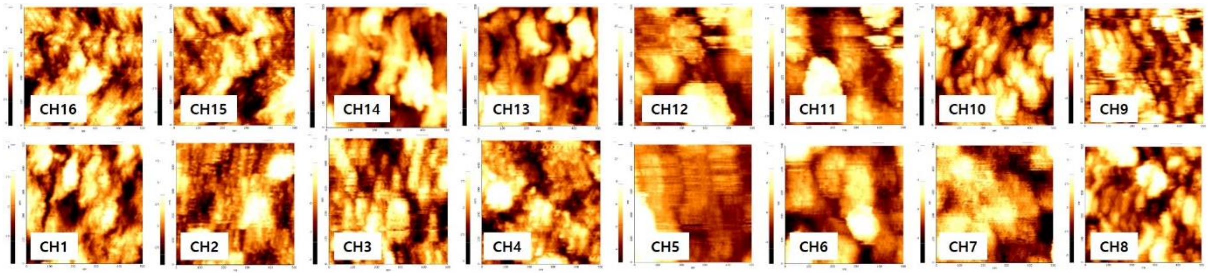

Figure S3. Top view AFM images of four metal oxide (SnO_2, TiO_2, WO_3 and In_2O_3) thin films. Image size: (a) 500 nm × 500 nm

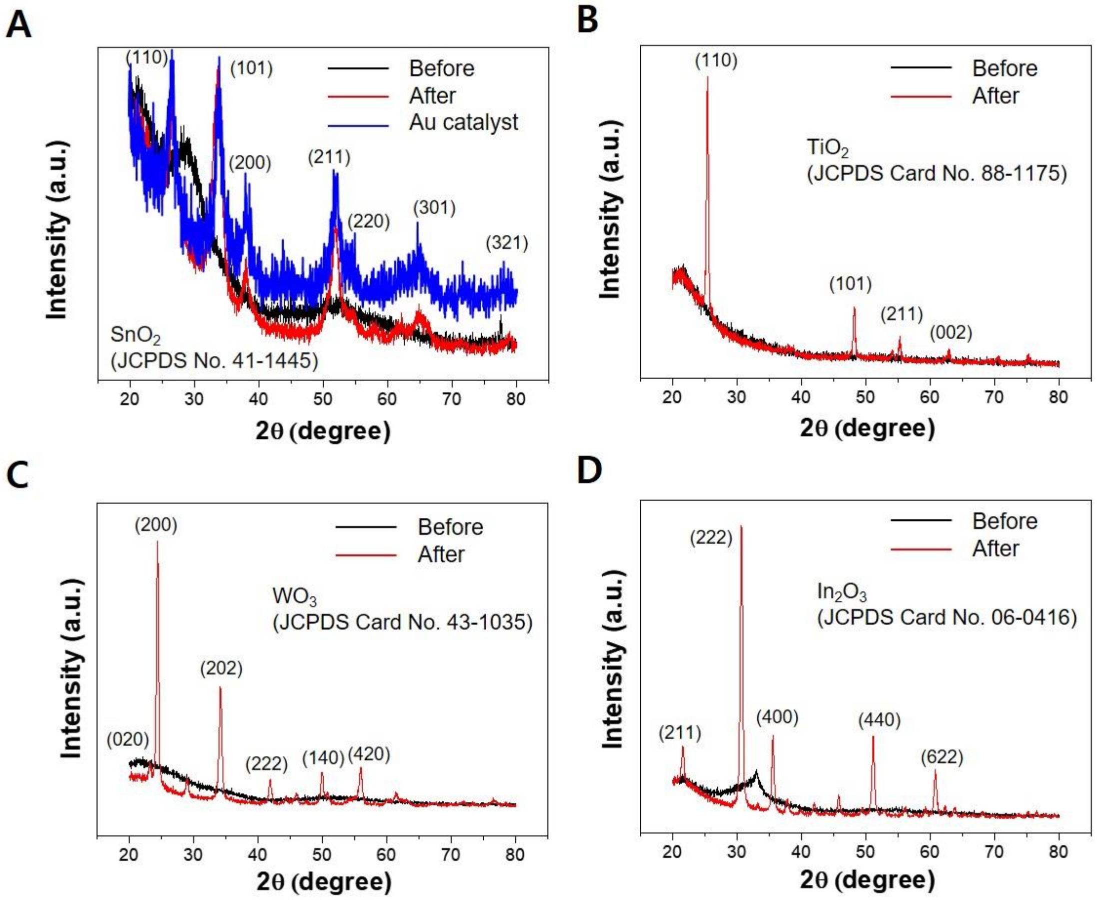

Figure S4. XRD patterns of four metal oxides (SnO_2, TiO_2, WO_3 and In_2O_3) thin films and an Au-functionalized SnO_2 thin film.

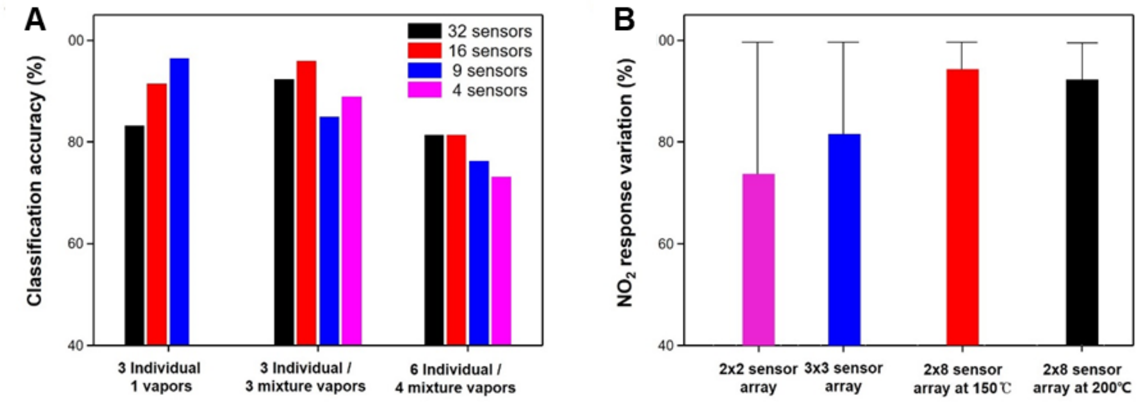

Figure S5. Configuration dependence of target classification. (A) Classification accuracy chart for individual vapors and vapor mixtures. (B) Variation of response amplitudes to NO_2 of different array configurations at different temperatures (2×2 sensor array: nanostructured SnO_2, nanostructured WO_3, nanostructured SnO_2+Au(catalyst), nanostructured WO_3+Au(catalyst) / 3×3 sensor array: SnO_2 thin film, In_2O_3 thin film, WO_3 thin film, SnO_2 thin film+Au(catalyst), In_2O_3 thin film+Au(catalyst), WO_3 thin film+Au(catalyst), nanostructured SnO_2, nanostructured In_2O_3, nanostructured WO_3 / 2×8 sensor array: SnO_2 thin film, WO_3 thin film, In_2O_3 thin film, TiO_2 thin film, SnO_2 thin film+Au(catalyst), WO_3 thin film+Au(catalyst), In_2O_3 thin film+Au(catalyst), TiO_2 thin film+Au(catalyst), SnO_2 thin film+Pt(catalyst), WO_3 thin film+Pt(catalyst), In_2O_3 thin film+Pt(catalyst), TiO_2 thin film+Pt(catalyst), SnO_2 thin film+Pd(catalyst), WO_3 thin film+Pd(catalyst), In_2O_3 thin film+Pd(catalyst), TiO_2 thin film+Pd(catalyst)).

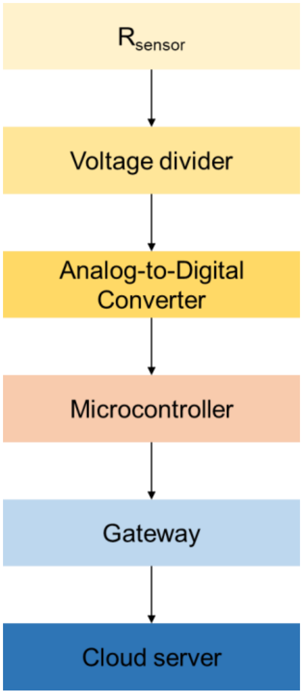

Figure S6. System level overview of the signal transduction, conditioning, processing, and wireless transmission.

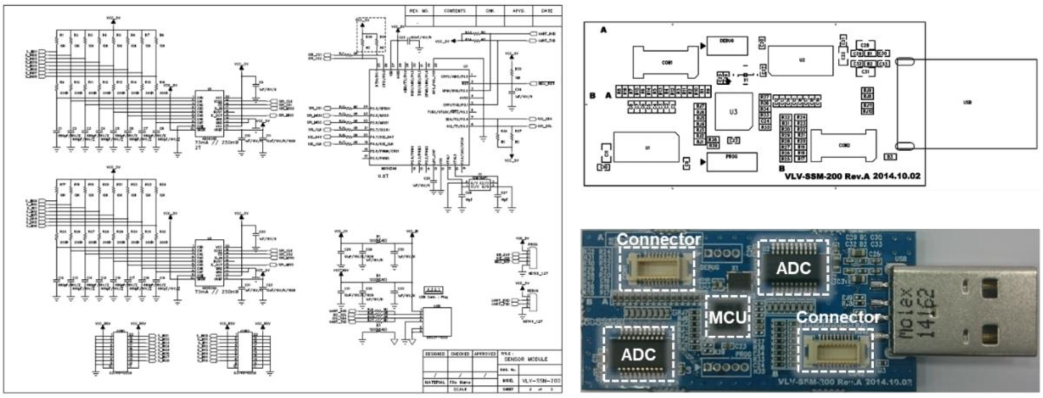

Figure S7. Schematics and photograph of the CSA module (bottom). Schematics of circuit components in the CSA module with a USB-type interface. The CSA was placed on a heater unit whose power consumption was 250 mW. The PCB was designed in such a way that the CSA was thermally isolated from other circuit components. The upper board with the CSA and the heater unit was plugged into the lower board containing a microcontroller unit and a 16-bit ADC for signal processing.

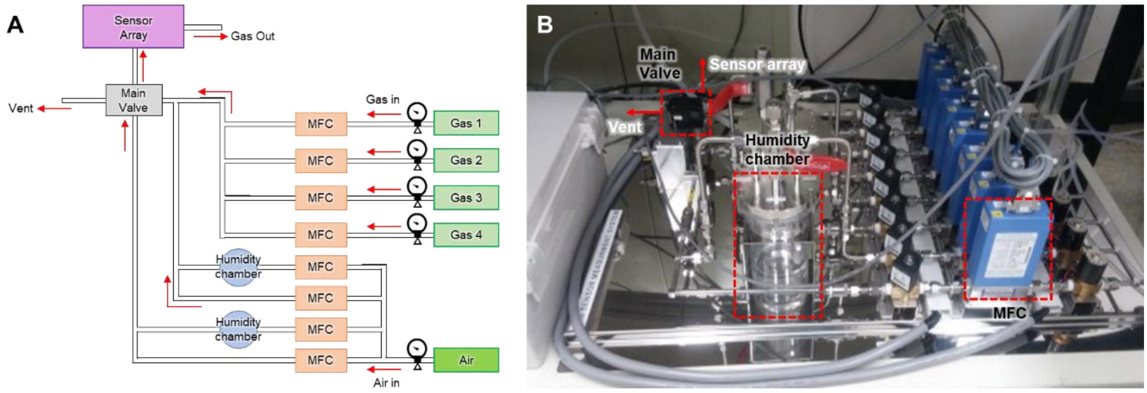

Figure S8. Gas flow and humidity control system for sensor characterization. (A) Schematic and (B) photograph image of the gas control system.

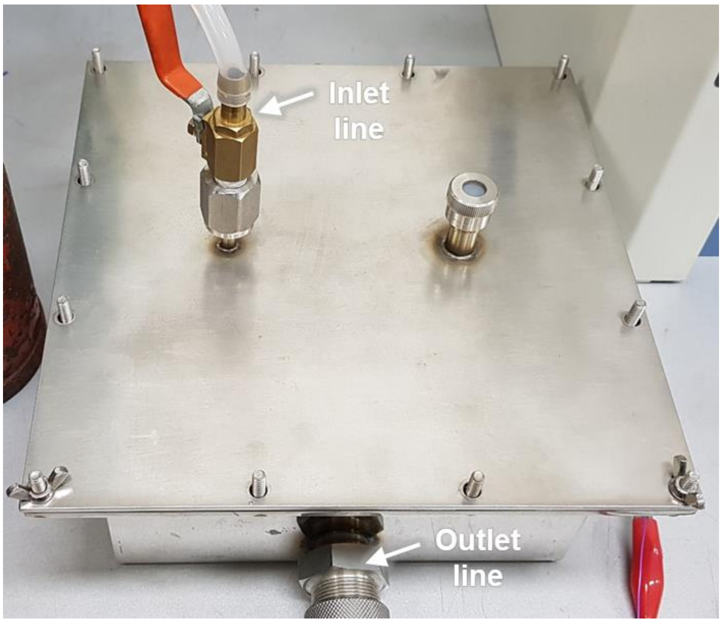

Figure S9. Measurement chamber for sensor characterization.

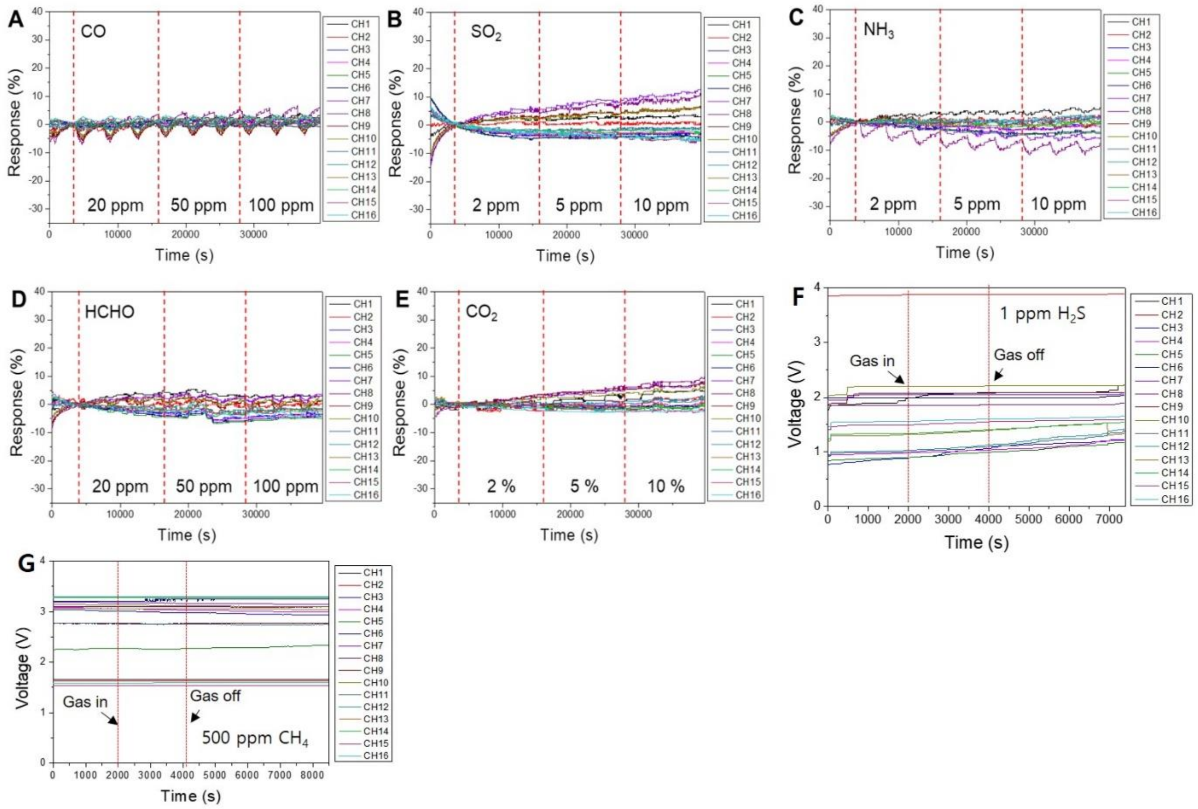

Figure S10. Continuous recording of the sensor responses to different chemical vapors. Typical sensor responses to (A) CO, (B) SO_2, (C) NH_3, (D) HCHO , (E) CO_2, (F) CH_4, and (G) H_2S.

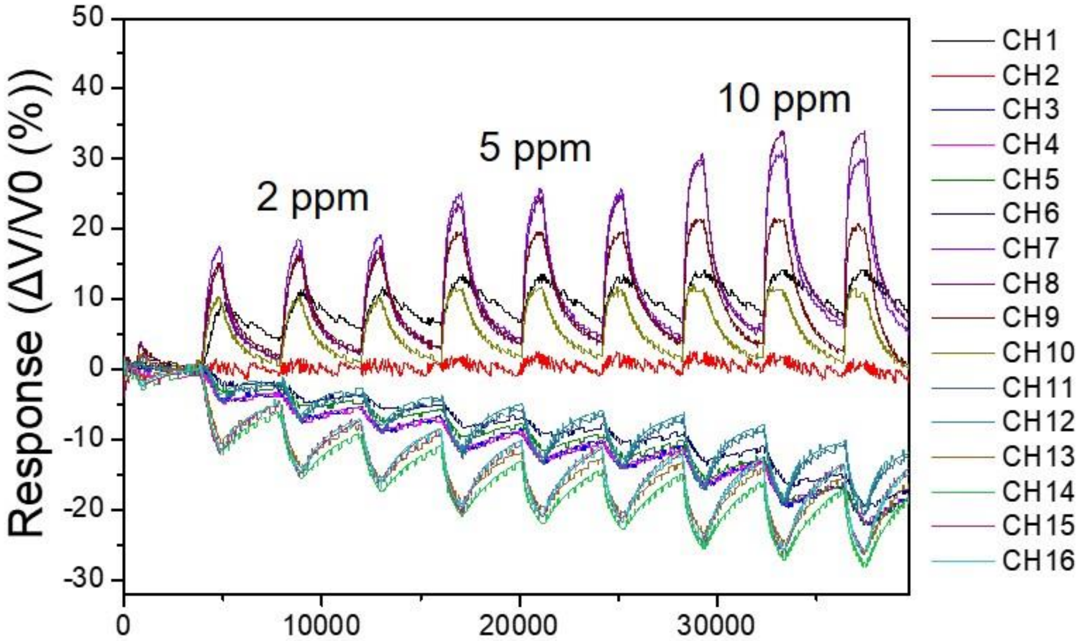

Figure S11. Responses for all channels to NO_2 vapors at concentrations of 2–10 ppm.

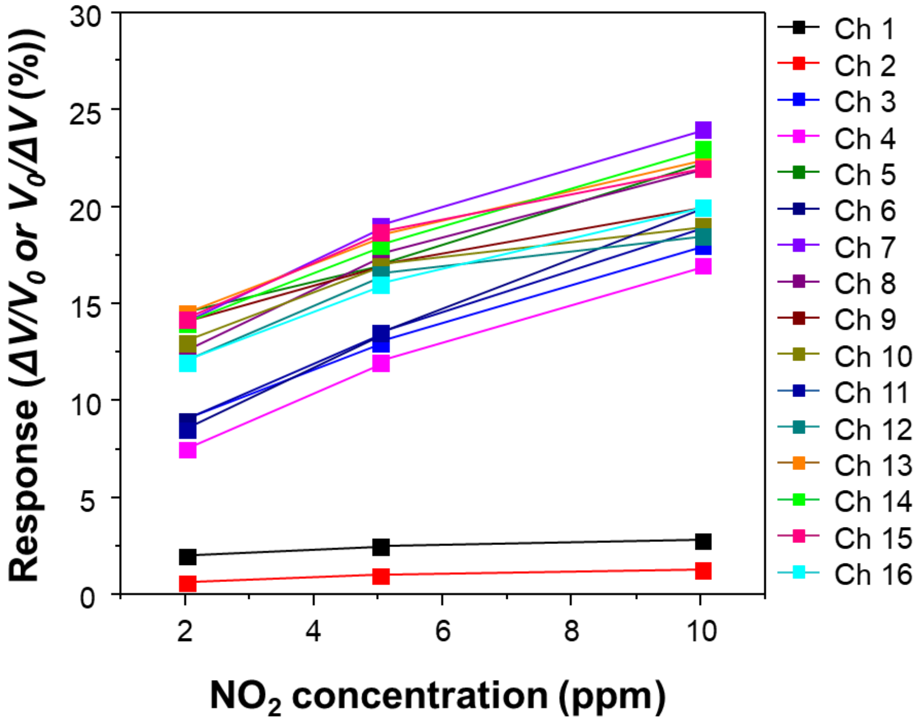

Figure S12. Responses of CSA at different NO_2 vapor concentrations in dry air at 180 °C. The theoretical detection limit for NO_2 (except for Channel 1 and 2) was evaluated to be in the range of 609–896 ppt by linear extrapolation.

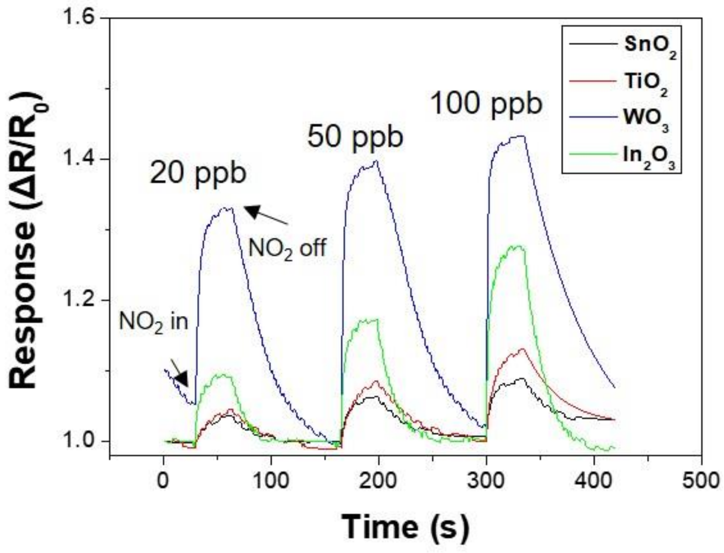

Figure S13. Real time response curves of four channels (1, 3, 5, and 7) in response to NO_2 vapors in the concentration range of 20–100 ppb at 180 °C.

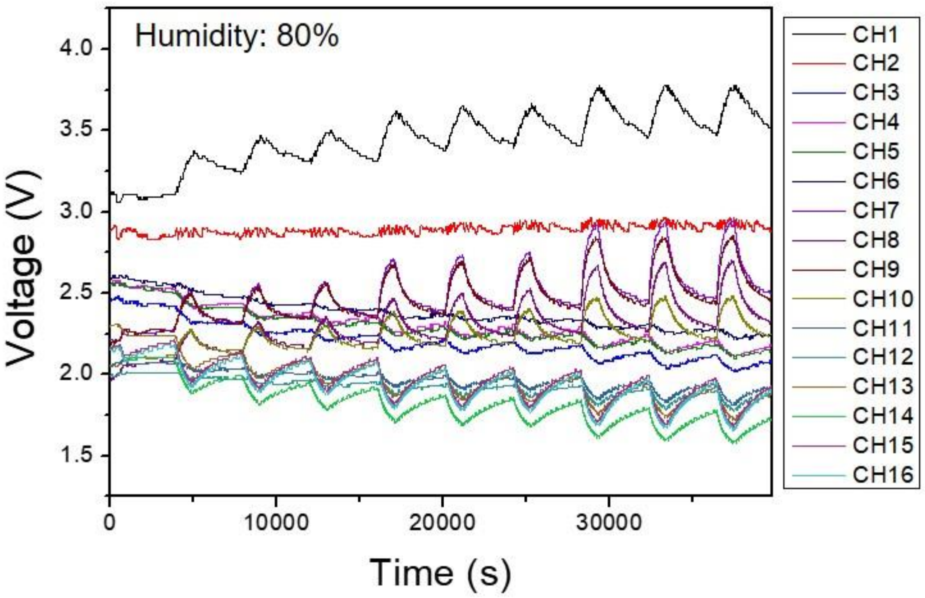

Figure S14. Real time response curves in response to NO_2 vapors in the concentration range of 2–10 ppm in a 80% relative humidity condition.

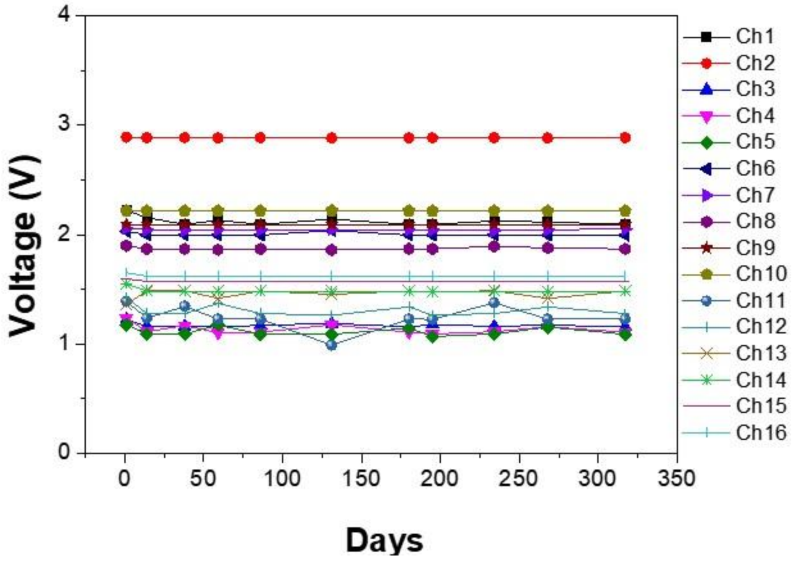

Figure S15. Long-term stability of all the channels in the CSA under exposure to the air for 317 days.

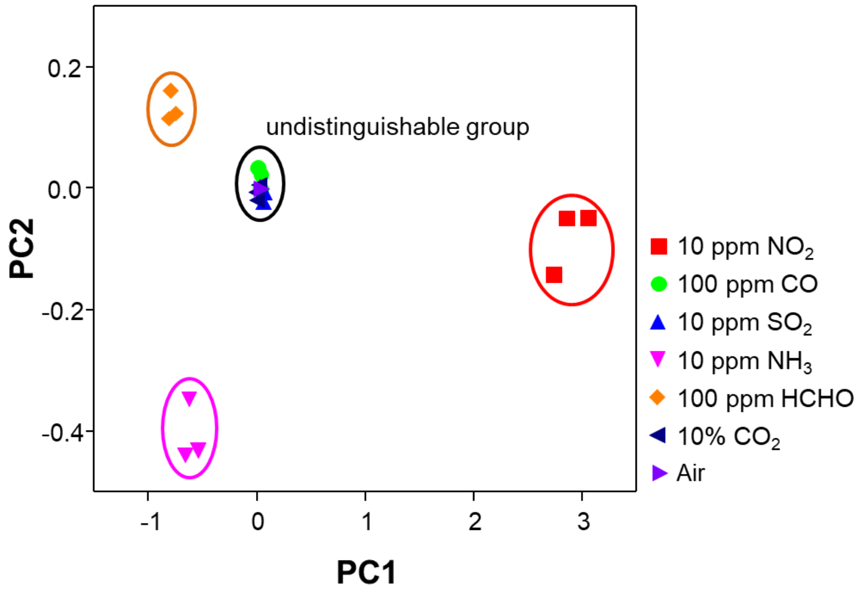

Figure S16. Principal component analysis (PCA) using the normalized responses of CSA. NO_2, NH_3, and HCHO vapors are well grouped in the PCA plot unlike other vapors.

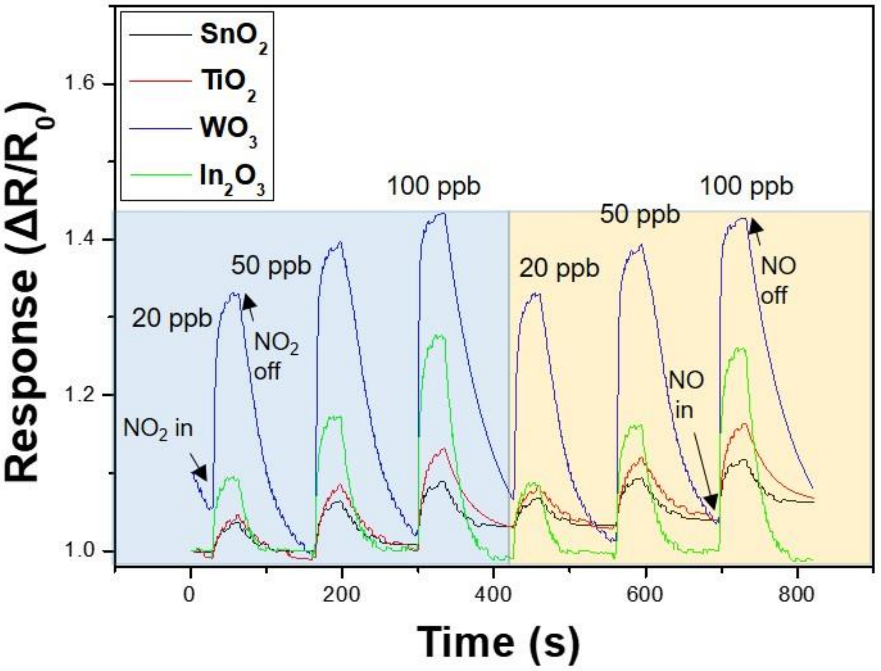

Figure S17. Responses of the CSA to NO and NO_2 vapors at concentrations of 20, 50, and 100 ppb.

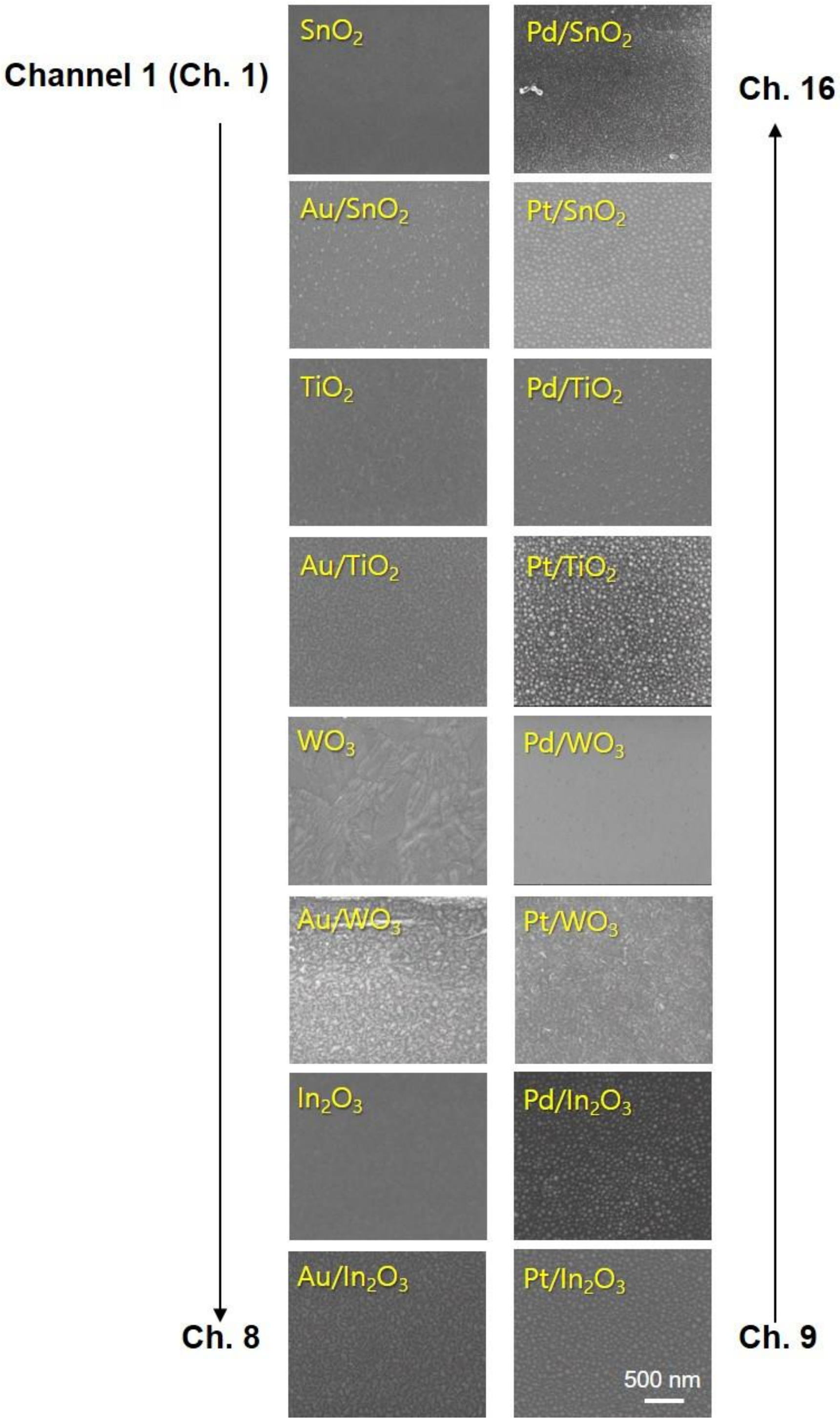

Figure S18. Field-emission scanning electron microscopy (FE-SEM) images of 16 sensor elements. After thermal processes, the functionalized layer of the novel metals formed nanoscale metallic islands.

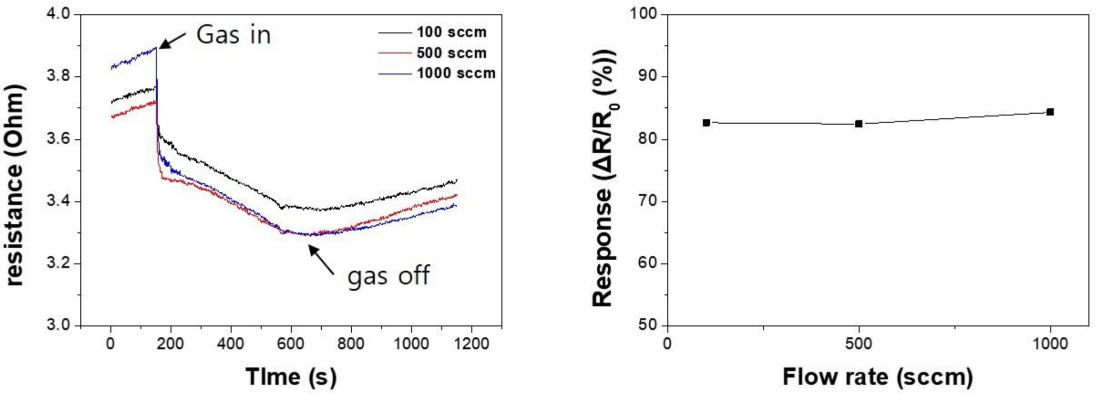

Figure S19. Real-time response curves in response to 500 ppm CO vapor at 400 °C with various mass flow controller (MFC) flow rates ranging from 100 to 1000 cm^3/min.

Table S1. Theoretical detection limits of sensor elements for NO_2 vapor.

Channel number	**rms**	**Slope**	**DL(ppm)**
**3**	0.00042	1.7568	0.000717
**4**	0.00046	1.6	0.000863
**5**	0.00047	1.7424	0.000809
**6**	0.00041	1.6547	0.000743
**7**	0.000397	1.59	0.000749
**8**	0.000409	1.69351	0.000725
**9**	0.00052	1.7405	0.000896
**10**	0.00047	1.6567	0.000851
**11**	0.00055	1.8644	0.000885
**12**	0.000355	1.748	0.000609
**13**	0.000435	1.69	0.000772
**14**	0.00051	1.7462	0.000876
**15**	0.00037	1.428	0.000777
**16**	0.000436	1.518	0.000862

To calculate the detection limit (DL), we applied equations to the obtained data as described below.

$V_{x^2} = \sum(y_i - y)^2$ (1)

where y_i is a measured value and y is the corresponding value obtained from the polynomial fit.

The rms noise is calculated as

$rms_{noise} = \sqrt{\frac{V_{x2}}{N}}$ (2)

where N is the total number of data points. The sensor noise is estimated using the amplitude of the variation from the baseline in the response curve. For example, the sensor noise of Channel 6 with Au-functionalized WO_3 to NO_2 is 0.00041.

$DL\ (ppm) = 3\frac{rms}{slope}$ (3)

With the definition of DL in IUPAC as shown above, the DLs for each channel are calculated.

For instance, with the slope of 1.6547 for Channel 6 its DL for NO_2 is 0.000743 ppm.

Table S2. Evaluation of binding affinities. Association rate constant (k_a), dissociation rate constant (k_d) and association constant (K_A) of Channel 7-10 sensors for NO_2 vapor.

	**Channel 7**	**Channel 8**	**Channel 9**	**Channel 10**
**k_a (10^5 $M^{-1}S^{-1}$)**	2.986±0.6872	0.2565±0.0055	0.8494±0.1792	0.8144±0.1405
**k_d (10^{-4} s^{-1})**	16.1±1.6643	13.7±0.8144	20.5±1.8681	20.7±1.6371
**K_A (10^9 M^{-1})**	0.1855	0.0188	0.0414	0.0393

Table S3. Comparison of commercial microheater-based gas sensor with our CSA.

Products	Power consumption (mW or mW/mm^2)
Figaro 260	350 mW
MiCS-5135 VOC Sensor	120 mW
Sentech Korea STK400	500 mW
Ogam GSBT11	250 mW
C_2H_5OH gas sensor[1]	350 mW/mm^2
Micro gas sensor[2]	550 mW/mm^2
MEMS microheaters-based gas sensor[3]	680 mW/mm^2

[1] S. E. Moon, H. K. Lee, N. J. Choi, J. Lee, C. A. Choi, W. S. Yang, J. Kim, J. J. Jong, D. J. Yoo, *Sens. Actuators, B* **2013**, 187, 598.
[2] J. Kang, J. S. Park, K. B. Park, J. Shin, E. A. Lee, S. Noh, H. J. Lee, *Micro and Nano Syst. Lett.* **2017**, 5, 26.
[3] Q. Zhoua, A. Sussman, J. Chang, J. Dong, A. Zettl, W. Mickelson, *Sens. Actuators, A* **2015**, 223, 67.
